# GLP-1–gut microbiotal axis and enteral feeding intolerance in critically ill neurological patients

**DOI:** 10.3389/fmed.2026.1807813

**Published:** 2026-03-30

**Authors:** Wei Li, Jie Zhang, Jingyan Pu, Qiuhong Pan, Li Chen, Hua Sui, Jiang Du, Zhimin Li

**Affiliations:** 1 Department of Nursing, Jiading Branch of Shanghai General Hospital, Shanghai Jiao Tong University School of Medicine, Shanghai, China; 2 Shanghai General Hospital, Shanghai Jiao Tong University School of Medicine, Shanghai, China; 3 Medical Experiment Center, Jiading Branch of Shanghai General Hospital, Shanghai Jiao Tong University School of Medicine, Shanghai, China; 4 Department of Critical Care Medicine, Jiading Branch of Shanghai General Hospital, Shanghai Jiao Tong University School of Medicine, Shanghai, China

**Keywords:** dysbiosis, enteral feeding intolerance, GLP-1, gut microbiota, neurocritical patients

## Abstract

**Introduction:**

Autonomic dysfunction following central nervous system injury predisposes critically ill neurological patients to enteral feeding intolerance (FI), but the underlying gut microbiota–glucagon-like peptide-1 (GLP-1) mechanisms remain unclear.

**Methods:**

We conducted a prospective cohort study of 60 patients in the intensive care unit receiving enteral nutrition (EN), with fecal and blood sample collection at admission (Q stage) and at the FI onset or EN cessation (H stage). The gut microbiota was analyzed via 16S rRNA sequencing, and GLP-1 and blood glucose were measured. Alpha and beta diversity, taxonomic composition, and correlations with GLP-1/glucose were evaluated.

**Results:**

At the Q stage, the tolerant group exhibited higher alpha diversity (ACE, Chao indices) and enrichment of *Blautia*, while intolerant patients showed early dysbiosis. At the H stage, beta diversity differed significantly (ANOSIM: R = 0.2, *P* = 0.01), with decreased *Bacteroidetes* and increased *Enterococcus* in intolerant patients. Spearman analysis revealed progressive strengthening of gut microbiotal correlations with GLP-1 and blood glucose, establishing a “gut microbiota–GLP-1–blood glucose” regulatory axis.

**Conclusion:**

Stage-dependent gut microbiotal changes predict the FI risk and correlate with GLP-1–mediated glucose and motility regulation. Early alpha diversity and specific taxa (e.g., *Bilophila*) may serve as predictive biomarkers. Targeting the gut microbiota and GLP-1 pathways represents a promising therapeutic strategy to improve EN tolerance in critically ill neurological patients.

## 1 Introduction

Autonomic dysfunction following central nervous system (CNS) injury is a key mechanism underlying enteral feeding intolerance (FI) in critically ill neurological patients. Impaired autonomic regulation can delay gastric emptying and disrupt intestinal motility, contributing to FI incidence rates of 38.3%–75% (1). Clinically, FI manifests as abdominal distension, gastric retention (residual gastric volume > 200 mL/6 h), and diarrhea, which reduce the efficacy of enteral nutrition (EN) and are associated with a prolonged hospital stay and increased mortality (2).

Glucagon-like peptide-1 (GLP-1), an incretin hormone, regulates glucose homeostasis by promoting insulin secretion and suppressing glucagon release, while also modulating gut motility. GLP-1 may influence gut microbiotal composition, increasing beneficial taxa such as *Lactobacillus* and *Bifidobacterium*, thereby enhancing intestinal barrier function (3, 4). Intensive care unit (ICU) studies suggest that GLP-1 improves EN tolerance by altering microbial composition (e.g., increasing *Bacteroides* and reducing *Enterococcus*) and decreasing plasma diamine oxidase and inflammatory cytokines (5).

Gut dysbiosis—reduced diversity and overgrowth of opportunistic microbes—is increasingly recognized as a key driver of FI (6). However, most studies focus on neonates (7), with limited data on neurocritical care patients, who are prone to autonomic dysfunction, vagal inhibition, and characteristic gut dysbiosis, often exhibiting reduced species abundance and simplified microbial communities (8). Evidence regarding dynamic microbiotal changes during hospitalization and their interactions with FI and GLP-1 remains limited.

We established a “GLP-1–gut microbiota–FI” framework, collecting dual-time point fecal samples at admission (Q stage) and at FI onset or EN cessation (H stage). Using 16S rRNA high-throughput sequencing and peripheral GLP-1 measurements, we state that the study aimed to explore the potential associations between GLP-1, gut microbiota modulation, and the development of FI and to inform potential probiotic or GLP-1–targeted interventions to improve nutritional tolerance and patient outcomes.

## 2 Materials and methods

### 2.1 Study design and participants

This prospective cohort study enrolled critically ill neurological patients receiving EN in the ICU at the Jiading Branch of Shanghai General Hospital from February 2024 to February 2025. The sample size was estimated based on the primary study endpoint, namely the detection of differences in gut microbiota alpha diversity between the FI and tolerant groups, with reference to the reported 38.3%–75% incidence of enteral feeding intolerance in neurocritical patients (1). A total of 60 patients were planned for enrolment to ensure adequate statistical power for this exploratory analysis. Patients were classified as tolerant (non-FI) or intolerant (FI) based on European Society of Intensive Care Medicine criteria, specific diagnostic parameters based on the ESICM criteria have been included, namely gastric residual volume (GRV) > 200 mL in two consecutive 6-h measurements, an increase in abdominal circumference > 2 cm within 24 h due to persistent distension, and recurrent diarrhea (≥3 loose stools per day) unrelated to antibiotics or other medications. Fecal and blood samples were collected at two time points: at 24 h after admission (Q stage) and at FI onset or EN cessation (H stage). All patients received a standardized EN formula (protein:fat:carbohydrate = 18%:30%:52%; energy density = 1.0 kcal/mL) with an initial infusion rate of 50 mL/h, gradually titrated to 60–80 mL/h according to clinical tolerance, with no differences between groups.

### 2.2 Inclusion and exclusion criteria

We included patients aged ≥ 18 years receiving EN support. We excluded patients with gastrointestinal disorders, malignant neoplasms, prior antibiotic/probiotic therapy, GLP-1 receptor agonist use, or early study termination owing to unforeseen circumstances.

### 2.3 16 S rRNA gene sequencing

Fecal samples were collected into sterile tubes and stored at −80 °C. DNA was extracted using the E.Z.N.A.^®^ Stool DNA Kit (Omega Bio-tek, Inc., GA, United States). The V3–V4 region of the 16S rRNA gene was amplified using specific primers on an EasyCycler 96 PCR system under defined cycling conditions, and sequencing was performed on an Illumina NextSeq 2000 platform. All samples were processed using identical protocols by the same personnel to ensure experimental consistency and minimize technical variability. Amplicon sequence variants (ASVs) were generated using DADA2, and taxonomic assignment was performed with the SILVA SSU138 database. Alpha diversity indices (ACE, Chao, Shannon, Simpson) and beta diversity (weighted UniFrac, ANOSIM) were assessed. Linear discriminant analysis Effect Size (LEfSe) identified differentially abundant taxa (LDA threshold = 3.0). The raw sequencing data of the 16S rRNA gene V3–V4 regions and accompanying information are available in the NCBI Sequence Read Archive database under accession number PRJNA1406443.

### 2.4 GLP-1 and blood glucose assays

Fasting venous blood (3 mL) was collected in ethylenediaminetetraacetic acid tubes and centrifuged; GLP-1 was quantified via enzyme-linked immunosorbent assay. Blood glucose was concurrently measured.

### 2.5 Statistical analysis

SPSS 26.0 (IBM Corp., Armonk, NY) was used for statistical analyses. Normally distributed data are expressed as mean ± SD and compared using *t*-tests; non-normal data are presented as median (interquartile range) and compared with Mann–Whitney U tests. Categorical data were compared using chi-squared tests. Spearman correlation evaluated relationships among the gut microbiota, GLP-1, and blood glucose. Significance was set at *P* < 0.05.

### 2.6 Ethics

The study was approved by the Ethics Committee of Jiading Hospital, Shanghai General People’s Hospital. Written informed consent was obtained from patients or their guardians.

## 3 Results

### 3.1 Baseline characteristics

Sixty patients with cerebral infarction (27, 45.0%), cerebral hemorrhage (26, 43.3%), subarachnoid hemorrhage (4, 6.7%), and traumatic brain injury (3, 5.0%) were enrolled. The mean age was 71.2 ± 14.6 years. Forty-nine patients (81.7%) were tolerant, and 11 (18.3%) were intolerant. Baseline demographics, comorbidities, blood glucose, and GLP-1 levels were balanced between the groups (all *P* > 0.05). ICU length of stay was longer in the intolerant group (*P* < 0.001) (Table 1).

**TABLE 1:** Comparison of clinical characteristics in neurocritical care patients.

Variable	Intolerant group (*n* = 11)	Tolerant group (*n* = 49)	*P*
Age (years)	71.91 ± 11.32	71.08 ± 15.31	0.867
Male (*n*, %)	6 (54.55%)	32 (65.31%)	0.747
Duration of ICU stay (days)	19 (12, 34)	10 (4.5, 14)	**<0.001**
BMI, kg/m^2^	23.5 (21.8, 26.6)	23.7 (22, 26.5)	0.819
Hypertension	7 (63.64%)	32 (65.31%)	0.916
Mechanical ventilation	5 (45.46%)	29 (59.19%)	0.621
Diabetes	7 (63.64%)	24 (48.98%)	0.586
Blood glucose (Q), mmol/L	10.01 (5.6, 12.58)	8.4 (6.55, 10.35)	0.606
GLP-1 (Q), pg/ml	748.44 (100.28, 1222.22)	154.31 (69.37, 730.75)	0.181
GCS score (Q)	8 (5, 9)	8 (5, 10)	0.985
Blood glucose (H), mmol/L	8.6 (5.2, 10.4)	6.75 (5.7, 7.67)	0.579
GLP-1 (H), pg/ml	536.38 (104.56, 1024.78)	146.61 (77.55, 963.22)	0.498
GCS score (H)	9 (7, 9)	8 (5, 10)	0.707

Values are expressed as mean ± SD or median (interquartile range) or number (%). *P* < 0.05 was deemed statistically significant. BMI, body mass index; GCS, Glasgow Coma Scale; GLP-1, glucagon-like peptide-1; H, collection point at the feeding intolerance onset or enteral nutrition cessation; ICU, intensive care unit; Q, collection point at 24 h after admission. Bold value indicates *P* < 0.05.

### 3.2 Gut microbiota diversity

At the Q stage, the tolerant group had higher alpha diversity (ACE: *P* = 0.013; Chao: *P* = 0.012). No differences were observed at the H stage (Table 2). Beta diversity (ANOSIM) was non-significant at the Q stage (*R* = 0.157, *P* = 0.065) (Supplementary Figure 1a) but was significant at the H stage (*R* = 0.2, *P* = 0.01), indicating dynamic shifts in microbial composition (Supplementary Figure 1b).

**TABLE 2:** Comparison of α diversity in the gut microbiota of critically ill neurological patients.

Alpha diversity metrics	Within 24 h of admission		*P*	FI onset/EN termination		*P*
	Tolerant group (QN)	Intolerant group (QP)		Tolerant group (HN)	Intolerant group (HP)	
ACE	123.65 (86.99, 149.82)	65.69 (45.20, 105.15)	**0.013**	97.71 (62.23, 128.52)	72.27 (45.00, 100.86)	0.106
Chao	123.32 (88.50, 149.83)	65.13 (45.00, 105.00)	**0.012**	96.80 (62.50, 128.75)	71.83 (45.00, 100.81)	0.119
Shannon	3.17 (2.78, 3.55)	2.49 (1.73, 3.19)	0.064	2.76 (2.25, 3.29)	2.32 (1.73, 2.55)	0.067
Simpson	0.09 (0.06, 0.16)	0.16 (0.08, 0.28)	0.124	0.12 (0.07, 0.24)	0.17 (0.15, 0.27)	0.168

Alpha diversity in the groups. *P* < 0.05 was deemed statistically significant. EN, enteral nutrition; FI, feeding intolerance; H, collection point at the feeding intolerance onset or enteral nutrition cessation; Q, collection point at 24 h after admission. Bold values indicate *P* < 0.05.

### 3.3 Microbial composition

At the phylum level, intolerant patients exhibited higher Bacillota (70.3% vs. 54.3%) and lower Pseudomonadota (2.6% vs. 9.5%; *P* = 0.035) levels at the Q stage. At the H stage, Bacteroidetes levels were reduced in intolerant patients (9.6% vs. 19.6%; *P* = 0.036).

At the genus level, tolerant patients were enriched with *Blautia* (3.27% vs. 2.81%; *P* = 0.031) at the Q stage and *Anaerococcus* (3.53% vs. 0.24%; *P* = 0.024) at the H stage. *Enterococcus* was consistently higher in intolerant patients (Table 3). LEfSe identified 11 Q-stage (Supplementary Figure 2a) and 8 H-stage microbial biomarkers (Supplementary Figure 2b), highlighting stage-specific microbial signatures linked to FI.

**TABLE 3:** Temporal dynamics of the dominant gut microbiota in neurocritical care patients.

Taxonomic level	Within 24 h of admission		*P*	At FI onset/EN termination		*P*
	Tolerant group (QN)	Intolerant group (QP)		Tolerant group (HN)	Intolerant group (HP)	
Genus level						
*Enterococcus*	3.41%	21.57%	0.119	21.55%	28.29%	0.564
*Bacteroides*	5.60%	3.39%	0.177	8.16%	7.42%	0.388
*Corynebacterium*	4.93%	0.50%	0.516	5.81%	6.29%	0.835
*Finegoldia*	7.20%	4.99%	0.253	5.02%	1.03%	0.119
*Escherichia-Shigella*	6.48%	1.71%	0.063	3.18%	7.62%	0.185
*Bifidobacterium*	6.41%	4.48%	0.691	3.14%	7.50%	0.968
*Blautia*	3.27%	2.81%	**0.031**	2.71%	4.53%	0.734
*Anaerococcus*	4.46%	3.53%	0.264	3.53%	0.24%	**0.024**
*Prevotella*	5.86%	10.01%	0.605	3.49%	0.14%	0.109
Others	52.68%	47.01%	0.053	43.41%	36.94%	0.259
Phylum level						
Bacillota	54.32%	70.25%	0.083	58.60%	54.65%	0.674
Bacteroidota	20.13%	18.68%	0.519	19.60%	9.56%	**0.036**
Actinomycetota	0.15%	7.03%	0.079	11.36%	14.79%	0.914
Pseudomonadota	9.46%	2.63%	**0.035**	7.99%	19.87%	0.194
Verrucomicrobiota	0.98%	0.01%	0.215	1.45%	0.13%	0.190
Campylobacterota	1.38%	0.49%	0.103	0.48%	0.08%	0.319
Fusobacteriota	0.36%	0.82%	0.933	0.32%	0.57%	0.675
Thermodesulfobacteriota	0.14%	0.06%	0.157	0.15%	0.31%	0.242
Synergistota	0.05%	0.02%	0.736	0.04%	0.03%	0.764
Others	13.03%	0.01%	0.545	0.01%	0.01%	0.614

*P* < 0.05 was deemed statistically significant. EN, enteral nutrition; FI, feeding intolerance; H, collection point at the feeding intolerance onset or enteral nutrition cessation; Q, collection point at 24 h after admission. Bold values indicate *P* < 0.05.

### 3.4 Correlations with GLP-1 and blood glucose

Spearman correlation revealed two ASVs at the Q stage and 12 at the H stage significantly associated with GLP-1 or blood glucose, demonstrating progressive intensification of microbiota–host interactions. For instance, *Bacteroides* (H stage) positively correlated with GLP-1 (*r* = 0.340, *P* = 0.008), while *Faecalibacterium* showed an inverse correlation with GLP-1 (*r* = –0.274, *P* = 0.034) but a positive correlation with glucose (*r* = 0.398, *P* = 0.002), suggesting disease-specific modulation of microbial function (Table 4).

**TABLE 4:** Spearman correlation analysis between gut microbiotal ASVs and GLP-1/blood glucose.

Grouping	ASV ID and genus	Blood glucose, mmol/L		GLP-1, pg/ml	
		*r*-value	*P*-value	*r*-value	*P*-value
QN-QP	ASV77 (*Bilophila*)	–0.017	0.894	–0.270	0.037
QN-QP	ASV155 (*Mobiluncus*)	–0.257	0.047	–0.021	0.873
HN-HP	ASV3 (*Clostridia*_UCG-014_Incertae_Sedis)	–0.047	0.722	–0.303	0.019
HN-HP	ASV78 (*Limosilactobacillus*)	0.060	0.650	–0.323	0.012
HN-HP	ASV114 (*Anaerovoracaceae*_unclassified)	–0.077	0.559	0.346	0.007
HN-HP	ASV183 (*Enterocloster*)	0.326	0.011	–0.105	0.427
HN-HP	ASV212 (*Bacteroides*)	–0.036	0.786	0.340	0.008
HN-HP	ASV353 (*Phocea*)	0.006	0.963	0.360	0.005
HN-HP	ASV414 (*Muribaculaceae*_Incertae_Sedis)	–0.078	0.554	–0.369	0.004
HN-HP	ASV726 (*Muribaculaceae*_Incertae_Sedis)	–0.082	0.535	–0.368	0.004
HN-HP	ASV876 (*Megasphaera*)	0.337	0.008	–0.080	0.542
HN-HP	ASV992 (*Candidatus Soleaferrea*)	–0.316	0.014	–0.122	0.352
HN-HP	ASV1124 (*Faecalibacterium*)	0.398	0.002	–0.274	0.034
HN-HP	ASV1200 (*Porphyromonas*)	0.091	0.491	–0.382	0.003

*P* < 0.05 was deemed statistically significant. ASV, amplicon sequence variant; GLP-1, glucagon-like peptide-1.

## 4 Discussion

This study demonstrates that stage-dependent gut microbiotal alterations are closely linked to FI development in critically ill neurological patients (9). Early alpha diversity and the presence of beneficial taxa such as *Blautia* and *Bilophila* may predict the FI risk (6, 10, 11), while subsequent dysbiosis (e.g., reduced *Bacteroidetes*, elevated *Enterococcus*) reflects the pathological progression of FI (12, 13). These findings are consistent with those of previous reports of initial microbial richness protecting against EN intolerance (6, 10).

The “gut microbiota–GLP-1–blood glucose” correlational axis identified here provides insights into the potential pathogenesis of FI. CNS injury may be associated with altered vagal activity (14) and hypothalamic-pituitary-adrenal axis function (15), which correlates with modified gut microbiota-driven GLP-1 stimulation. Reduced GLP-1 is associated with compromised glucose homeostasis and intestinal motility, which correlates with an increased risk of FI (16). Once FI occurs, impaired nutrient absorption and barrier function are associated with exacerbated dysbiosis, reflecting a potential correlational cycle: dysbiosis → GLP-1 abnormality → FI → further dysbiosis (17).

Therapeutically, our findings highlight multiple intervention targets. Probiotic supplementation of taxa such as *Blautia* or *Bacteroides* is associated with restored microbial diversity (18, 19) and enhance short-chain fatty acid production, which correlates with improved GLP-1 secretion and intestinal motility (20). GLP-1 receptor agonists are associated with potential improvements in EN tolerance and metabolic regulation (21, 22). Monitoring early microbial diversity and specific taxa may allow proactive identification of patients at high risk for FI, enabling timely interventions to improve nutritional delivery and clinical outcomes (18, 23).

The sample size of the FI group in the present study was relatively small (*n* = 11), which may have compromised statistical power and limited our ability to detect subtle differences and weak correlations between microbial composition and FI. This limitation may also have increased the risk of Type II errors (false negatives). Therefore, our findings should be interpreted with caution and will require confirmation in future large-scale, multicenter cohort studies. This study exhibits clinical heterogeneity in H-stage sample collection: the intolerance group underwent symptom-triggered sampling at the onset of feeding intolerance, whereas the tolerance group underwent treatment-endpoint-triggered sampling at EN cessation. This difference may have obscured microbial signatures specifically associated with feeding intolerance. In future studies, fixed-time serial sampling protocols will be implemented to minimize this potential bias. In addition, this study is limited by uncontrolled potential confounders. Future studies should incorporate multivariable regression analyses to better account for confounding factors and to clarify the associations between gut microbiota, GLP-1, and FI.

In conclusion, within the “GLP-1–gut microbiota–FI” framework, this study showed that early gut microbiotal diversity and specific taxa (e.g., *Bilophila*) predict the FI risk in critically ill neurological patients. Overall, dynamic alterations in microbial composition correlate with GLP-1 and blood glucose regulation, reflecting a potential axis linking CNS injury to FI. These findings provide novel insights into the pathogenesis of FI and suggest potential therapeutic directions targeting gut microbiota modulation and GLP-1 signaling. However, these associations require further validation before clinical application, particularly with regard to improving EN tolerance and patient outcomes. Validation in larger, multicenter studies is warranted.

## Data Availability

The datasets presented in this study can be found in online repositories. The names of the repository/repositories and accession number(s) can be found below: https://dataview.ncbi.nlm.nih.gov/object/PRJNA1406443?reviewer=slve95gm2nstgst3rp3j2d57bv, PRJNA1406443.
