# Supplementary material for: GLP-1–gut microbiotal axis and enteral feeding intolerance in critically ill neurological patients
Source: Front Med (Lausanne). 2026 Mar 30;13:1807813. doi: 10.3389/fmed.2026.1807813 (PMC13070781; doi:10.3389/fmed.2026.1807813)
Supplement: Supplementary file 1 [file Table_1.docx]

Supplementary Material

**
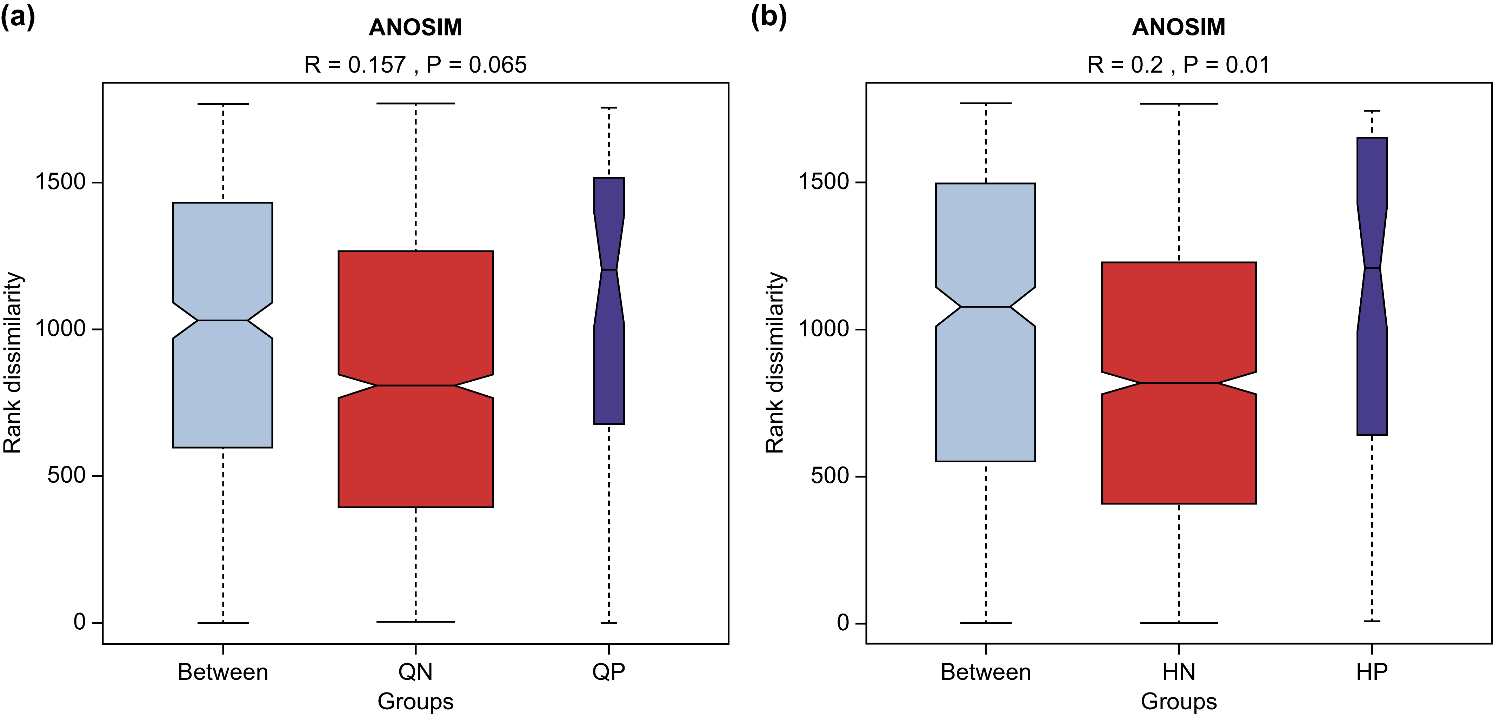
**

**Supplementary Figure 1.** Beta-diversity of the gut microbiota in the tolerant and intolerant groups at the Q and H stages. (**A**) Weighted UniFrac-based beta-diversity analysis revealed no significant structural differences at the Q stage (ANOSIM: R = 0.157, P = 0.065). (**B**) At the H stage, the microbial communities differed significantly between the tolerant (HN) and intolerant (HP) groups (ANOSIM: R = 0.2, P = 0.01).

FI, feeding intolerance; H, collection point at the feeding intolerance onset or enteral nutrition cessation; Q, collection point at 24 h after admission


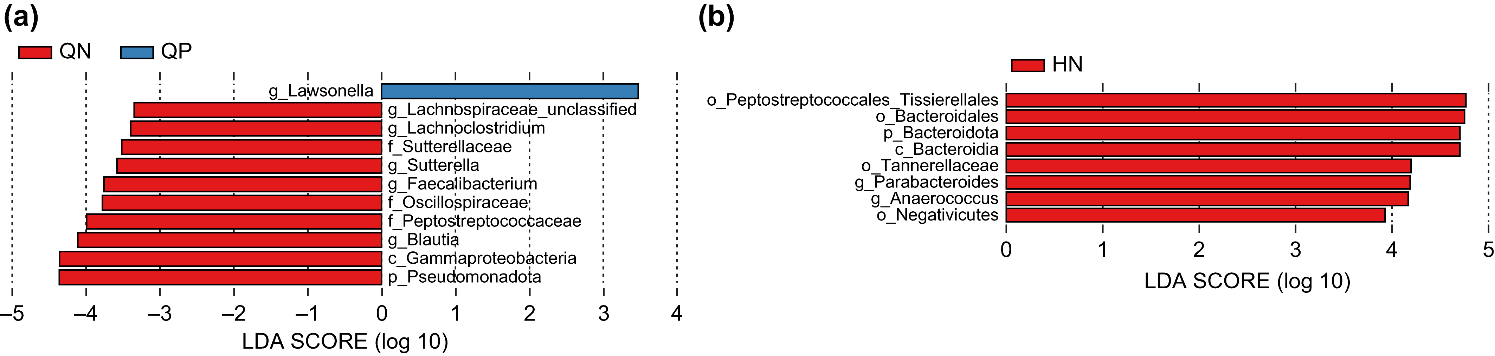


**Supplementary Figure 2.** Differential gut microbial biomarkers identified through LEfSe at the Q and H stages. LEfSe (LDA > 3.0) identified stage-specific microbial markers. (**A**). At the Q stage, the tolerant (QN) group was dominated by *Blautia*, *Faecalibacterium*, phylum Pseudomonadota, and class γ-Proteobacteria, whereas the intolerant (QP) group showed no dominant taxa. (**B**). At the H stage, the tolerant (HN) group was enriched in phylum Bacteroidota, class Bacteroidia, order Bacteroidales, and genera *Anaerococcus* and *Parabacteroides*, whereas the intolerant (HP) group was dominated by order Peptostreptococcales-Tissierellales.

EN, enteral nutrition; FI, feeding intolerance; H, collection point at the feeding intolerance onset or enteral nutrition cessation; LEfSe, linear discriminant analysis Effect Size; Q, collection point at 24 h after admission
